# Perception of Old Age in German Undergraduate Dental Students—A Comparison of Two Cohorts 10 Years Apart

**DOI:** 10.3390/ijerph18063279

**Published:** 2021-03-22

**Authors:** Sophia Weber, Ina Nitschke, Sebastian Hahnel, Angelika Rauch

**Affiliations:** 1 Clinic for Prosthetic Dentistry and Dental Materials Science, Leipzig University Medical Center, 04104 Leipzig, Germany or ina.nitschke@zzm.uzh.ch (I.N.); sebastian.hahnel@medizin.uni-leipzig.de (S.H.); angelika.rauch@medizin.uni-leipzig.de (A.R.); 2 Clinic of General, Special Care and Geriatric Dentistry, University of Zurich, 8006 Zurich, Switzerland

**Keywords:** attitudes toward aging, dental care for aged, geriatric dentistry, older adults

## Abstract

Previous studies revealed that students’ willingness to provide dental services for older patients is mainly influenced by their individual perception of elders rather than their knowledge about old age. The aim of this study was to estimate students’ perception of old and young age as well as their hopes and fears associated with old age and to compare two cohorts that participated in the study 10 years apart. Data were obtained from a questionnaire completed by two cohorts of undergraduate dental students from 2006 to 2008 (T1, *n* = 207) and 2016 to 2018 (T2, *n* = 135). Participants were asked to define the ages that they consider a man or woman to be old and young. Moreover, they had to specify their fears and hopes associated with old age. Reported thresholds for old age differed significantly between T1 and T2. In contrast to T1 students, T2 students defined a person to be old at a higher age and barely differentiated between the old ages of men and women. Furthermore, T2 students presented more fears related to aging than T1 students, e.g., psychological problems or loss of independence. The perception of age appears to be a multifactorial process and significantly changed between students of T1 and T2. Fears of dental students regarding old age should be addressed in, e.g., gerodontological curricula, to foster positive experiences in interaction with older people and highlight the important and rewarding aspects of gerodontology.

## 1. Introduction

Demographic changes are leading to a shift in the population pyramid; in 1997, only 15.8% of the German population were older than 65 years, while there was an increase to 21.4% in 2018 [1]. According to the United Nations, an older person is defined as “a person who is over 60 years of age” [2], whereas the most important German epidemiological study on oral health refers to older people when reaching the age of 65 years [3]. The number of older adults is still rising as high birth rates in Germany from 1950 to 1970 are leading to more people who are now between 50 and 70 years old [4]. In addition, advances in modern medicine and introduction of innovative therapy options against lethal communicable diseases have caused an increase in lifespan [5].

At the same time, intensive oral health promotion measures in Germany have led to less edentulous patients within older people [3,6]. According to recent extrapolations, by 2030, the prevalence of edentulism in Germans aged between 60 and 80 years will drop to 4.2% [7].

In Germany, statutory health insurance covers two dental examinations and one periodontal debridement per year. Today, patients show higher acceptance toward dental hygiene therapy and a noticeable awareness of the importance of individual oral hygiene measures practiced at home than in 2005 [8]. As a result, oral health-related problems, such as caries or periodontitis, are deferred to later stages in life when individuals are older and frailty increases [9], which leads to more complex dental treatments but potentially limited options of such [10]. Thus, dental practitioners are not only facing increased numbers of older people suffering from oral health-related problems, but also an increasing percentage of complex treatment decisions resulting from the preservation of teeth, the need for prosthetic rehabilitation at a higher age, and impaired general health of patients. This is especially true since previous studies reported self-perceived conflicts of dentists regarding subjective and objective treatment needs as well as for autonomy or beneficence [11].

There is a wide heterogeneity among older patients, and it is particularly pertinent that dentists can meet the variety of physical and psychological needs of the older and changing adult population. With regard to this aspect, it has been shown that empathy of dental practitioners relevantly improves the satisfaction of patients [12]. Previous investigations revealed that students’ willingness to provide healthcare for older individuals is primarily influenced by their individual perception of older people rather than their knowledge about old age [13]. Thus, positive attitudes toward older people must be taught and promoted in educational programs [14]. However, it is not only attitudes that might change with time and by exposure to geriatric issues—individual fears and hopes associated with old age [15] might also be influenced by images of older people as purported in the media or by personal encounters. Over five years, investigations have observed longitudinal changes of students’ perceptions of old age during their participation in an undergraduate gerodontology program [15]; however, changes have never been investigated over a prolonged period when comparing two different undergraduate cohorts that are questioned 10 years apart.

The current investigation aimed to estimate undergraduate dental students’ perceived thresholds for being old and young and to determine their fears and hopes associated with old age with the use of a questionnaire. Two cohorts (2006–2008 and 2016–2018) were separately interviewed in the course of their individual curriculum, and potential differences were identified and interpreted. The working hypotheses suggest no shift regarding the definition of being old and young between the two cohorts; and fears and hopes associated with old age did not change within a period of 10 years.

## 2. Materials and Methods

All undergraduate dental students taking part in their first gerodontology curriculum at the Department for Prosthodontics and Materials Science at the University of Leipzig were recruited for this study. The curriculum is biannually implemented in the context of prosthodontic dental courses at the University of Leipzig even if it is not required according to the regulations for the licensing of dentists. It includes a one-day clinical course in a long-term care facility for older people, where each student is visiting one resident of the facility. In addition to taking medical history, students perform an extra- and intraoral examination and inspect dental prostheses. Students are requested to conduct minor dental treatments that are possible within the facility such as periodontal debridement or adjustment of the dental prostheses. After the visit, each student completes an essay about the particular patient, including a treatment recommendation and its coordination. As a part of the gerodontological education program in Germany and Switzerland, questionnaires made of multiple-choice questions were developed by one of the authors (I.N.), specialized in the field of gerodontology. During various educational programs in gerodontology, questionnaires were handed to students in Zürich (Switzerland) and Leipzig (Germany). In Leipzig, questionnaires were handed to all participating students in order to evaluate and coordinate the gerodontological curriculum. The regulations of the study protocol, which assure protection of data privacy and confidentiality during analysis of questionnaires, were explained to all participants in writing before receiving the subjects’ consent. There were no bonus-malus regulations for participants. Questionnaires were filled out anonymously, disabling withdrawal from the study later on.

For this study, survey data of first-time participants without previous contact to older patients in a professional context were analyzed. The data were obtained from an anonymous questionnaire completed just before entering the curriculum. The first cohort to answer the questionnaire was recruited from 2006 to 2008 (T1). The second cohort included students from courses between 2016 and 2018 (T2). In the first part of the questionnaire (which was identical in the two cohorts), the age and sex of the undergraduate students were recorded, and participants were asked to define the age threshold when they consider a man or a woman to be old and young. The second part of the survey addressed students’ fears and hopes associated with old age. A choice of modified responses according to Roux [16] was given, and multiple answers were possible. In this study, questionnaires and the acquisition of data were performed in accordance with a previous study that addressed a similar research approach and methodology surveying an undergraduate student population between 2004 and 2009 [15]. The study design and questionnaire were evaluated by the local Ethics Committee, indicating that no study approval was necessary.

Statistical analyses (IBM SPSS Statistics 25.0.0.0, IBM, Armonk, NY, USA) were conducted to describe demographic characteristics of both cohorts. Since no normal distribution was identified (Shapiro–Wilk test: *p* ≤ 0.001), age definitions in T1 and T2 were tested for significance using the Mann–Whitney test. Correlation between the age of the participating students and their definitions of being old and young was determined with Pearson correlation. The Pearson correlation coefficient (r) was interpreted as weak (r = 0.2–0.5), moderate (r = 0.51–0.7), strong (r = 0.71–0.9), or very strong (r > 0.9) [17]. Fears and hopes of students associated with old age were identified as percentages of positive responses for each item using the Chi-square test. For statistically significant differences of fears and hopes between T1 and T2, odds ratios (OR) were determined including 95% confidence intervals (CI). The level of significance was set to *p* ≤ 0.050.

## 3. Results

All recruited students completed the questionnaire, yet in some cases, a few data points were missing due to incomplete questionnaires. During the first period of observation (2006–2008, T1), 207 students were recruited. The mean age of the students was 22 years (±2.8 years; range: 20–38 years). Sex was distributed as 60.4% female (*n* = 125) and 38.2% male (*n* = 79), while 1.4% (*n* = 3) did not report their sex. Due to decreased enrollment of students in undergraduate courses, 135 students were recruited for the second observation period (2016–2018, T2) with a mean age of 24 years (±3.8 years; range: 21–43 years) including 69.6% women (*n* = 94) and 30.4% men (*n* = 41).

### 3.1. Definition of Age Limits

#### 3.1.1. T1 vs. T2 (Overall)

For the definition of old age (Table 1), students statistically significantly increased threshold ages for men from 60.6 years in T1 (±8.3 years; range: 40–80 years) to 65.7 years in T2 (±7.8 years; range 40–90 years; *p* < 0.001) and for women from 59.8 years in T1 (±9.2 years; range 35–80 years) to 65.1 years in T2 (±8.7 years; range 36–90 years; *p* < 0.001).

#### 3.1.2. T1 vs. T2 (Female Students)

For the definition of old age, female students statistically significantly increased threshold ages for men (T1/T2: 61.3/65.2 years; *p* ≤ 0.001) and women (61.0/65.0 years; *p* ≤ 0.001). A statistically significant difference was identified for the definition of being young as a man (35.3/38.3 years; *p* ≤ 0.023).

#### 3.1.3. T1 vs. T2 (Male Students)

For the definition of old age, male students statistically significantly increased threshold ages for men (T1/T2: 59.8/66.8 years; *p* < 0.001) and women (57.5/65.3 years; *p* < 0.001). There was no statistically significant difference in perception of young age between male students of T1 and T2.

### 3.2. Correlation of Students’ Age and Their Definition of Being Old/Young

In T2, there was a weak positive correlation between an increased age of the students and their definition of being old. In contrast to younger students, older students considered both men (r = 0.28, *p* ≤ 0.001) and women (r = 0.24, *p* ≤ 0.005) young for a longer period.

### 3.3. Fears and Hopes

#### 3.3.1. T1 vs. T2 (Overall)

In both T1 and T2, participants were mostly worried about sickness (T1/T2: 80.2/77.8%) and physical decline (65.7/71.9%) (Table 2). The most frequent hope of undergraduates was “time for the family” (74.9/74.1%) followed by “cultivating friendships” in T1 (56.5%) and “tranquility/serenity” in T2 (58.5%). Comparing the T1 and T2 cohort, there was a statistically significant increase in fears related to a “loss of independence” (41.1/53.3%; *p* ≤ 0.029; OR 1.63) and “psychological problems” (20.8/37.0%; *p* ≤ 0.001; OR 2.23); however, the fear of “no employment” statistically significantly declined (15.9/7.4%; *p* ≤ 0.019; OR 0.42). Regarding hopes, “being available for others” declined from T1 to T2 (16.9/6.7%; *p* ≤ 0.005; OR 0.35). Significant differences between T1 and T2 were only observed for female students.

#### 3.3.2. Female vs. Male Students (T1)

Sex-related analysis of the T1 cohort revealed statistically significant differences between male and female students in four categories. Male participants were less afraid of “sickness” (M/F: 67.1/88.0%, *p* < 0.001) but feared “psychological problems” more frequently (27.8/16.0%, *p* ≤ 0.037) and had fewer hopes of “being available for others” (7.6/22.4%, *p* ≤ 0.006) or of “starting a new life” (6.3/16.8%, *p* ≤ 0.031).

#### 3.3.3. Female vs. Male Students (T2)

In the T2 cohort, male students were less afraid of “losing their independence” (M/F: 34.1/61.7%; *p* ≤ 0.003) and had fewer hopes for “tranquility/serenity” (43.9/64.9%; *p* ≤ 0.023) when thinking about life as an older person.

## 4. Discussion

The results of this investigation revealed a statistically significant change in perception of old age and hopes and fears associated with old age when comparing the two cohorts (2006–2008 and 2016–2018) of undergraduate dental students. Thus, the working hypotheses of this study were rejected.

### 4.1. Definition of Age Limits

In the T2 cohort, the threshold for being old was postponed to higher ages (65 years), which agrees with data reported by other investigators [18,19], and threshold ages supported by the Fifth German Oral Health study of 2014, defining youngerseniors at age 65–74 [3]. However, definitions of old age did not correspond to the German legal retirement age of 67 years. The delayed thresholds between T1 and T2 appear as a relevant change in mindset and might be caused by a different perception of old age that could be triggered by different stereotypes purported in the media and by digitalization. Within recent years, increasing access to digital media has changed the way information can be accessed. Students who have commenced their studies at universities within the past few years grew up in a digital world [20]. As “digital natives” [21,22], they are familiar with omnipresent access to the internet [23]. Thus, they have the opportunity to easily collect manifold information and draw a more differentiated and complex picture of age and the heterogeneity of the aging process. Moreover, the changed perceptions of old age might be due to a shift of the average German retirement age (T1/T2: 63.2/64.1 years) [24] and a higher global life expectancy, which increased from 61.7 years in 1980 to 71.8 years in 2015 [25]. The national data for Germany corroborate this trend and prove that life expectancy recently increased from 77.2 years (T1) to 78.5 years (T2) for men and from 82.4 years (T1) to 83.3 years (T2) for women [26]. Additionally, perception of old and young age might also be influenced by individual factors, such as the family background or the place of residence. A shift in the definition of old age might have an impact on the dentists’ subconscious perception of their patients, which might relevantly affect the way they are treated in dentistry. Whereas in the T1 cohort, a 60-year-old male patient was defined as old, students of T2 defined a later threshold. Consequently, T1 dentists might have a different opinion on the patients’ physical and cognitive abilities. This might result in differences in decision making, such as choosing therapy options for dental modifications or repair. Regarding the definition of young age, there were no significant differences within the overall group. Thus, it appears that the threshold for being young is more constant than for being old. Moreover, this observation might also be explained by the similar ages of the participating students in T1 and T2.

When investigating the outcome of the survey regarding its dependence on sex, female students did not set different age thresholds for women and men, while male students set the threshold for being old lower in women than in men. This phenomenon is described as the “double standard of aging” [27,28] and indicates that men are more critical toward the aging process of the opposite sex [29,30,31]. In addition, the earlier onset of the female biological maturation process might result in a different perception of social norms compared to those of male classmates. In this study, although male participants distinguished between sexes, an attenuation of the effect was observed: the thresholds for old age in women and men showed a disparity of 2.3 years in T1, but this decreased to 1.5 years in T2. This observation could be attributed to a growing awareness of gender equality in society, politics, and economics in recent years, which has been increasingly promoted by governmental facilities such as the German “Federal Ministry for Family Affairs, Senior Citizens, Women and Youth” [32] or by university degree programs [33].

### 4.2. Correlation of Students’ Age and Their Definition of Being Old or Young

Results revealed a weak positive correlation between increasing age of undergraduate dental students and their definition of old age, with a statistical significance in T2. Since the mean age of participants in T2 was slightly higher than in T1, this phenomenon might be explained by a more differentiated perception of age by the older students in T2.

### 4.3. Fears and Hopes

An analysis of students’ fears associated with old age revealed that the dominating fears, which were declared by approximately two thirds of the participants in both cohorts, were primarily related to physical aspects of aging such as “sickness” and “physical decline”. Because younger adults often describe themselves as physically active and healthy, a potential loss of these characteristics might induce fears. The most uncommon fear in both cohorts was “no employment”. This observation was not surprising given the fact that the mean age of participants was 22 (T1) and 24 (T2) years, which indicates that the participants were at the beginning of their studies and had their whole working career ahead of them. Moreover, the percentage of this fear significantly declined from 15.9% in T1 to 7.4% in T2, which might be explained by the comprehensive German healthcare system that provides basic dental treatments to all Germans and therefore secures continuous and almost complete employment of dental professionals. This is corroborated by data of the German “Federal Employment Agency”, which revealed that only 1% of German dentists declared unemployment in 2018 [34]. In addition, the participants of the T2 cohort grew up in a period of prosperity without dramatic economic crises.

However, comparisons of fears related to old age between the T1 and the T2 cohort revealed a significant increase toward “psychological problems” and “loss of independence”. This might be due to an increase in the number of diseases characterized by psychological problems, such as dementia—a condition that affects 50 million people worldwide, which is expected to increase to 82 million in 2030 and 152 million in 2050 [35]. Furthermore, it is likely that new generations are more prone to psychological distress and major depression episodes than before [36], which might be due to the negative influence of the excessive use of electronic devices [37]. In general, students’ increasing awareness of psychological issues, which was observed in the present investigation, might foster the implementation of psychological screening tools in dental education [38].

The primary hope of students in both cohorts was “time for family”, followed by “cultivating friendships” (T1) and “tranquility/serenity” (T2). As digitalization increases, the ubiquitous use of digital media and communication are dramatically accelerating contemporary lifestyles, and people tend to answer emails/phone calls or finish working tasks in their free time. Thus, it has been reported that younger people have a growing desire for a healthier work–life balance [39,40,41].

Interestingly, significant changes in fears and hopes between T1 and T2 were solely observed for female participants. Women might be more affected by external factors than their male classmates, e.g., by psychological diseases or the ideal of women as purported in the media. Increased fears regarding old age can also raise concerns as they might lead to biased attitudes toward older individuals and may influence student–patient relationships [19,42]. Thus, favorable experiences in interactions with older adults should be promoted [13,43]. In dentistry, curricula dealing with care of older people that are designed for undergraduate students can help to highlight the important and rewarding aspects of gerodontology and sensitize for the aging process by improving attitudes of the students [15,44].

The limitations of the current study include the minor age range of participants. Comparing age definitions and the perception of old age, older participants might have a different view on age-related topics. However, it can be assumed that the perception of age was influenced by the peer group rather than by biological age, since the small number of older undergraduates were exposed to similar influences to those of their younger classmates. Moreover, it would be interesting to identify undergraduate dental students’ knowledge about aging or aging processes, which could help to correlate their attitudes. This might be performed with lectures on aging and age-related topics, e.g., medical conditions of older people and consequences for dental treatment. A written exam or an objective structured clinical examination (OSCE) could be used to reveal students’ knowledge and clinical competencies. In addition, students’ personal experiences with older adults could be considered for further investigations. Since the questionnaire was primarily designed as a tool to evaluate and coordinate the educational aspect of the gerodontology curriculum rather than to be used as a research instrument, it should only be discussed in this context.

## 5. Conclusions

The results of this investigation revealed that the attitudes of dental undergraduate students regarding old age changed from 2006–2008 to 2016–2018. The threshold for being old shifted to higher ages. Compared to the T1 cohort, male students of the T2 cohort revealed less distinction between age thresholds for men and women. Moreover, there was a significant increase in fears associated with psychological problems and the loss of independence. Gerodontological education programs might be a helpful tool in dental undergraduate education to address students’ fears regarding old age. Identifying those fears and thematizing them with the students might help to eliminate concerns and consequently foster positive experiences and impart skills for dental treatment of older patients.

## Figures and Tables

**Table 1:** Thresholds for being old and young as issued by undergraduate dental students in two cohorts (2006–2008 (T1), 2016–2018 (T2)); significant differences between T1 and T2 are presented in bold; missing data due to incomplete questionnaires.

		T1 (2006–2008)		T2 (2016–2018)		T1 vs. T2
Participants	Question	*n*	Mean (SD) Years	*n*	Mean (SD) Years	*p*-Value (Mann Whitney-Test)
Overall	♂ old	204	60.6 (8.3)	134	65.7 (7.8)	**<0.001**
	♀ old	204	59.6 (9.2)	133	65.1 (8.7)	**<0.001**
	♂ young	203	34.9 (7.7)	133	37.1 (10.4)	n.s.
	♀ young	203	34.6 (7.7)	133	36.8 (10.6)	n.s.
Female Participants	♂ old	122	61.3 (7.8)	93	65.2 (7.4)	**0.001**
	♀ old	122	61.0 (7.7)	93	65.0 (8.1)	**0.001**
	♂ young	121	35.3 (5.9)	93	38.8 (11.1)	**0.023**
	♀ young	121	35.1 (6.5)	93	38.6 (11.3)	n.s.
Male Participants	♂ old	79	59.8 (8.9)	41	66.8 (8.6)	**<0.001**
	♀ old	79	57.5 (10.8)	40	65.3 (9.9)	**<0.001**
	♂ young	79	34.2 (9.8)	40	33.2 (7.5)	n.s.
	♀ young	79	33.6 (9.3)	40	32.6 (7.1)	n.s.

**Table 2:** Percentage (count) of changes in fears and hopes (modified according to Roux [16]) related to old age between T1 (2006–2008) and T2 (2016–2018) for all students and divided by sex (Chi-square test); ^†^ N (overall) = 206; sex was not reported by three participants; significant differences between T1 and T2 are presented in bold * and odds ratios (OR) including the 95% confidence intervals (CI) are presented.

		T1 (2006–2008)			T2 (2016–2018)			
		Overall *n* = 206 ^†^	Female *n* = 125	Male *n* = 78	Overall *n* = 135	Female *n* = 94	Male *n* = 41	OR (95% CI)
Fears	Sickness	80.2 (166)	88.0 (110)	67.1 (53)	77.8 (105)	78.7 (74)	75.6 (31)	-
	Physical decline	65.7 (136)	68.8 (86)	62.0 (49)	71.9 (97)	75.5 (71)	63.4 (26)	-
	Loss of close relatives	62.8 (130)	63.2 (79)	63.3 (50)	61.5 (83)	66.0 (62)	51.2 (21)	-
	**Loss of independence** *	41.1 (85)	44.0 (55)	36.7 (29)	53.3 (72)	61.7 (58)	34.1 (14)	1.63 [1.05; 2.52]
	Reduced activity	35.7 (74)	35.2 (44)	35.4 (28)	40.0 (54)	39.4 (37)	41.5 (17)	-
	Boredom	23.3 (48)	23.2 (29)	24.1 (19)	23.7 (32)	23.4 (22)	24.4 (10)	-
	**Psychological problems** *	20.8 (43)	16.0 (20)	27.8 (22)	37.0 (50)	39.4 (37)	31.7 (13)	2.23 [1.37; 3.62]
	Approaching death	20.3 (42)	23.2 (29)	13.9 (11)	25.9 (35)	27.7 (26)	22.0 (9)	-
	Social isolation	20.3 (42)	21.6 (27)	17.7 (14)	28.1 (38)	30.9 (29)	22.0 (9)	-
	**No employment** *	15.9 (33)	19.2 (24)	10.1 (8)	7.4 (10)	7.4 (7)	7.3 (3)	0.42 [0.20; 0.88]
	Others	2.4 (5)	2.4 (3)	2.5 (2)	1.5 (2)	1.1 (1)	2.4 (1)	-
Hopes	Time for the family	74.9 (155)	76.8 (96)	70.9 (56)	74.1 (100)	75.5 (71)	70.7 (29)	-
	Cultivating friendships	56.5 (117)	61.6 (77)	32.9 (38)	53.3 (72)	55.3 (52)	48.8 (20)	-
	Time for oneself	54.1 (112)	53.6 (67)	54.4 (43)	49.6 (67)	48.9 (46)	51.2 (21)	-
	Tranquility/serenity	53.1 (110)	52.8 (66)	51.9 (41)	58.5 (79)	64.9 (61)	43.9 (18)	-
	Retaining lifestyle	26.6 (55)	24.0 (30)	29.1 (23)	31.9 (43)	27.7 (26)	41.5 (17)	-
	Less obligations	25.6 (53)	21.6 (27)	32.9 (26)	31.9 (43)	29.8 (28)	36.6 (15)	-
	No need to work anymore	23.7 (49)	21.6 (27)	26.6 (21)	20.0 (27)	19.1 (18)	22.0 (9)	-
	To communicate	19.3 (40)	20.0 (25)	19.0 (15)	16.3 (22)	16.0 (15)	17.1 (7)	-
	**Being available for others** *	16.9 (35)	22.4 (28)	7.6 (6)	6.7 (9)	7.4 (7)	4.9 (2)	0.35 [0.16; 0.75]
	Starting a new life	12.6 (26)	16.8 (21)	6.3 (5)	13.3 (18)	13.8 (13)	12.2 (5)	-
	Others	3.4 (7)	4.8 (6)	1.3 (1)	3.7 (5)	2.1 (2)	7.3 (3)	-

## Data Availability

Data sharing is not applicable to this article.
